# Study on Lubrication and Friction Reduction Properties of ZIF-8 Nanoparticles as Si_3_N_4_ Ceramic Water Lubrication Additives

**DOI:** 10.3389/fchem.2021.802375

**Published:** 2021-12-20

**Authors:** Tianyi Sui, Lichao Li, Bin Lin, Yuhang Zhang, Benyang Zhang, Shuai Yan

**Affiliations:** Key Laboratory of Advanced Ceramics and Machining Technology of Ministry of Education, School of Mechanical Engineering, Tianjin University, Tianjin, China

**Keywords:** MOFs, lubricant additives, nanoparticles, superlubricity, ceramics

## Abstract

Ceramics can achieve superlubricity under water lubrication; however, their running-in period is long and application is rather limited by wear limit. Thus, zeolite imidazole ester skeleton (ZIF), an important branch of metal organic framework materials (MOFs), is expected to improve the tribological properties of lubricants and associated additives. As such, it has broad application prospects within the field. In this paper, ZIF-8 nanoparticles of varying concentrations were prepared and linked with amino functional groups. Specimens were used in silicon nitride self-matching pairs and their tribological properties were observed. After the experiment, friction surfaces were analyzed by scanning electron microscope (SEM), energy dispersive spectrometer (EDS), and Fourier transform infrared radiation (FTIR). The experimental results have shown that ZIF-8 nanoparticles greatly reduced both friction and wear. Comprehensively considering running-in time, average COF during the whole process and smooth friction period COF, optimal performance was obtained for the ZIF-8 nanoparticle solution concentration of 1wt%. Furthermore, it was concluded that the lubrication properties of amino-modified ZIF-8 nanoparticles are significantly better compared to that of the unmodified ZIF-8. The anti-friction mechanism of ZIF-8 as a ceramic water lubrication additive was mainly through the filling and forming of nanoparticle film on the ceramic surface.

## Introduction

The ceramic water lubrication has attracted much attention since reducing the friction coefficient below 0.002 results in the superlubricity stage. Hence, it significantly reduces both friction and wear, which is of great significance for energy conservation and reduction of emissions (Tomizawa and Fischer, 1987; Xu and Li, 2015). However, ceramic water lubrication requires a long running-in period before the superlubricity can be achieved. Additionally, the wear is rather severe, limiting the application of ceramic friction pairs (Duan et al., 2021; Edward Kennedy and Rajaram, 2021). In recent years, nanomaterials have shown great potential as lubricating additives (Spikes, 2015; Fuskele and Sarviya, 20172017), which was confirmed by an increasing number of studies and applications.

Among them, MOFs, as a novel nanomaterial, were widely used in hydrogen storage, drug delivery, catalytic reaction, biosensor, and gas adsorption and separation, mostly due to their high porosity and good chemical stability (Rosi et al., 2003; Rowsell and Yaghi, 2006; Banerjee et al., 2008; Xu et al., 2011; Wu et al., 2013). As an important branch, ZIF-8 is a porous material with zeolite topology, which has broad prospects in industrial application. It can be prepared by several methods (Matsukata et al., 1999; Tian et al., 2007; Zheng et al., 2010; Cravillon et al., 2011; Zhu et al., 2011; Seoane et al., 2012), with the thermal solvent method being the most advanced. Thermal solvent method is simple and product performance is stable, making it suitable for industrial application.

Previous studies have shown that adding the nanoparticles to the lubricant significantly improved its anti-wear and anti-friction performance. There are several lubricant additive working mechanisms, such as dispersively bearing the pressure (Peng et al., 2009), forming the protective surface film (Zhang et al., 2012), smoothing the surface (Sui et al., 2016), and transforming the sliding into rolling friction (Jiao et al., 2011). Additionally, it was found that ZIF-8 has very good chemical and thermodynamic stability, as well as strong gas adsorption (Li et al., 1999; Chen et al., 2001). When applied to lubricant additives, it has the potential to adsorb surface reaction products, forming a more stable protective layer. In addition, previous studies have shown that ZIF-8 nanoparticles, especially the amino-modified ones (Ni et al., 2021), can significantly improve the lubricant anti-friction and anti-wear effects, while also significantly shortening their running-in time (Ding et al., 2018; Cui et al., 2020). However, only few studies were focused on ZIF-8 and their modified nanoparticle solution, which could improve the lubricating performance of ceramic friction pairs.

For this reason, various concentrations of unmodified and amino-modified ZIF-8 nanoparticles were added to water-based lubricants. Further, tribological properties of silicon nitride friction pairs were tested for each concentration. The effect of ZIF-8 nanoparticles on the friction characteristics of ceramic water lubrication was studied, and the associated lubrication mechanism was analyzed based on the characterization results.

## Methods

In the experiment, the ZIF-8 methanol solution was prepared via the solvothermal method. The reaction speed was fast and the product crystallinity was good. The specific method was carried out as follows: the solution was stirred at room temperature using a JKI-TN-2 mini stirring table (provided by Shanghai Jingxue Scientific Instrument Co. Ltd.) at the speed of 400 r/min.

The friction experiment was carried out using the MMW-1 friction test machine, provided by Jinan Puye Electromechanical Technology Co. Ltd. Ball disk friction pairs made of silicon nitride were used in the experiment. After the tribological experiment, morphological characteristics such as wear scar diameter on the silicon nitride ball surface were observed and the working mechanism of ZIF-8 nanoparticles was analyzed. Further (3-aminopropyl) triethoxysilane purchased by Sinopharm Chemical Reagent Co. Ltd., was selected for the modification process.

Following the friction test, the surface was characterized via SEM and EDS using the Hitachi SU8010 scanning electron microscope. Additionally, friction surface was also characterized using the FTIR with Bruke infrared spectrometer. SEM was used to observe the surface micro-morphology, while EDS and FTIR detected elements and functional groups.

The preparation of ZIF-8 nanoparticles can be divided as follows: firstly, zinc nitrate hexahydrate (Zn (NO_3_)_2_·6H2O), 2-methylimidazole (2-mim), and methanol (MeOH) (purchased by Sinopharm Chemical Reagent Co. Ltd.) were placed into the reaction instrument. The molar ratio was 1:10:700.10 mmol (2.97 g) of Zn(NO_3_)_2_·6H_2_O were fully dissolved with 140 ml of methanol. Next, 100 mmol (8.21 g) of 2-methylimidazole were also dissolved with 140 ml of methanol. After the two solutions were completely dissolved, zinc nitrate hexahydrate methanol solution was stirred with magnetic rod. Next, the methanol solution of 2-methylimidazole was added into the methanol solution of zinc nitrate hexahydrate quickly. Once the solution dissolved completely, the solution was stirred using a magnetic rod at room temperature for 5 h. Next, the nanocrystals were separated from the emulsion dispersion by centrifugation and through fresh absolute ethanol washing. The obtained ZIF-8 nanocrystals were then dried in an oven at 40°C to obtain ZIF-8 particles in a white powder state (Ullah Khan et al., 2018; Wang et al., 2021).

**FIGURE 1:**
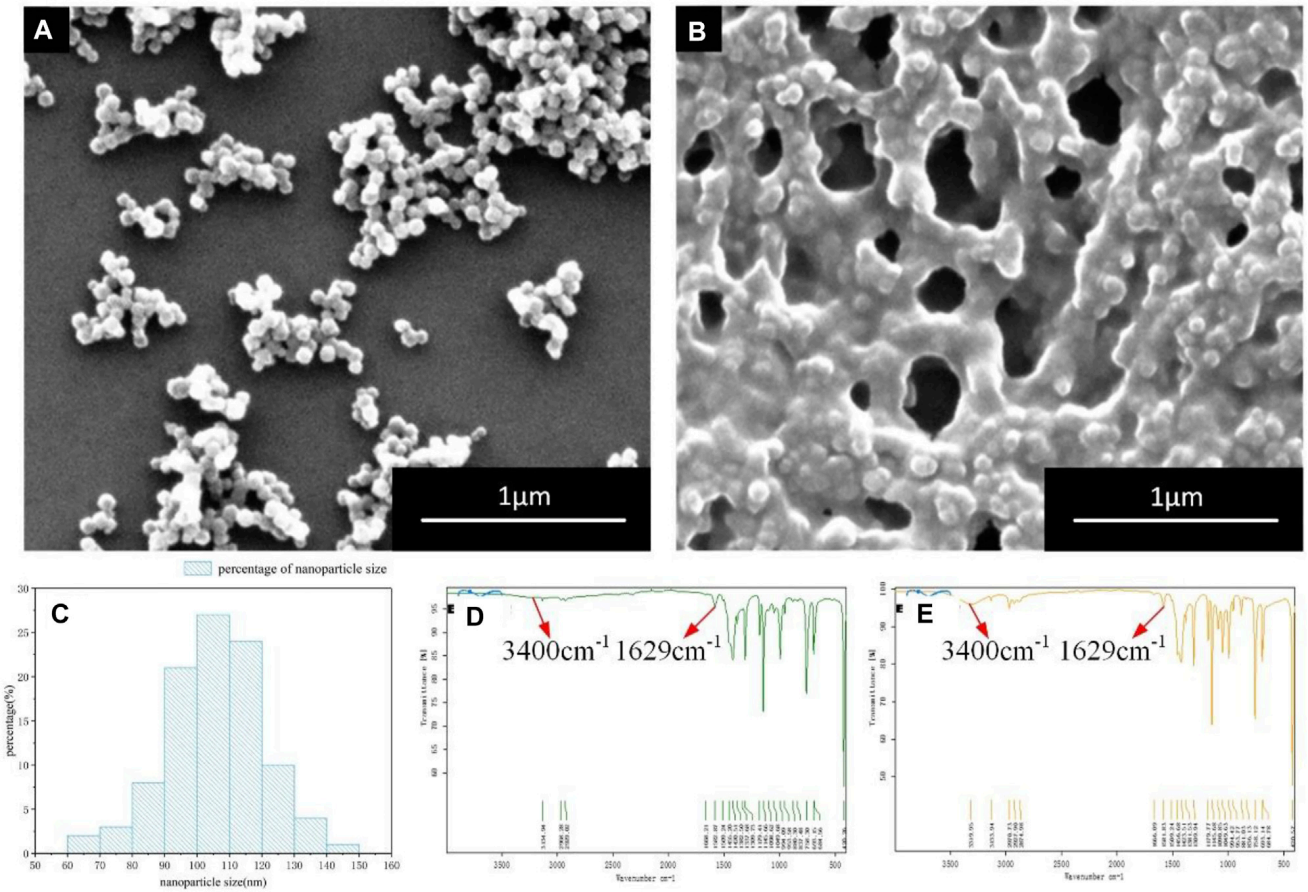
SEM and FTIR images of ZIF-8 nanoparticles before and after the amino modification **(A)** Unmodified ZIF-8 nanoparticles (SEM) **(B)** Amino-modified ZIF-8 nanoparticles (SEM) **(C)** fZIF-8 nanoparticle size distribution **(D)** Fourier infrared spectrum of ZIF-8 modified nanoparticles **(E)** Fourier infrared spectrum of ZIF-8 unmodified nanoparticles.

After preparation, absolute ethanol was added into the nanoparticle solution to dilute the sample solution to about 0.05%. After that, a drop of solution was dropped to the center of the silicon wafer. Next, the silicon wafer dripping with solution is sent to the oven for drying. After completing the above operations, the sample preparation could be observed under the scanning electron microscope. Because the conductivity of ZIF-8 nanoparticles is not strong, the direct observation effect can not get the required experimental results. In order to achieve better observation effect, it’s necessary to spray gold to increase its conductivity. The SEM image is shown in Figure 1A.

After the analysis of the SEM image shown in Figure 1A, it can be seen that the particle size of ZIF-8 was relatively uniform, approximately 100 nm. Moreover, the morphology was good and the ZIF-8 nanoparticles had a regular hexahedron shape (Santoso et al., 2021).

The organic skeleton on the surface of ZIF-8 nanoparticles contained a large number of hydroxyl groups that react with the silane coupling agent, connecting different functional groups to the ZIF-8 nanoparticle surfaces. Furthermore, in this experiment, the modifier selected was (3-aminopropyl) triethoxysilane, which could connect amino functional groups to the ZIF-8 nanoparticle surface, improving the adsorption on the friction surface.

The modification process was carried out and the specific process was listed as follows: Excessive (3-aminopropyl) triethoxysilane was added to ZIF-8 nanoparticle solution following the growth of nano ZIF-8 crystal. The stirring was continued for 6 h to ensure that the amino groups will be fully linked to the surface of ZIF-8 nanoparticles. After observing and analyzing the SEM images (Figure 1B), it was concluded that the amino groups were linked to the surface of ZIF-8 nanoparticles. Hence, after the amino modification, ZIF-8 nanoparticles were closely connected by amino functional groups, forming a quasi-dense structure with good adsorption characteristics, which could be easily adsorbed on the friction surface (Lin et al., 2019). After that, measurement of nanoparticle size was done. The measurement result is shown in Figure 1C. The particle size of most ZIF-8 nanoparticles was about 100 nm.

Subsequently, the FTIR was used to detect friction surfaces. The Fourier infrared spectra of both the modified and unmodified ZIF-8 nanoparticles were shown in Figure 1D, E. By comparing the two figures, it was observed that after modification, there were more peaks near points of 1,629 cm^−1^ and 3,400 cm^−1^. The additional peaks represented the N-H bond and N-H stretching vibration peak. On the other hand, there were no N-H bonds in ZIF-8; however, there was an obvious N-H chemical bond after modification, showing that the amino modification successfully linked the amino-functional groups on the surface of ZIF-8 nanoparticles.

The diameter of the ceramic ball used in the friction test was 9.525 mm and its Rockwell hardness was 78. In this experiment, the vertical loading pressure was 30 N, the spindle speed was 207 r/min, the speed was 0.5 m/s, the friction radius was 23.075 mm, and the experimental time was set to 3,600 s.

Upon completing the tribological experiment, the surface was analyzed using both the SEM and EDS, and surface morphology elements were detected. In addition, the wear scar diameters of the ceramic balls were measured by electron microscope. On the characterization image, the diameters of wear spots were measured in four directions (horizontal, vertical, +45° , −45° ) using measurement software. After the measurement, the average of the four diameters was taken as the final result of the measurement.

## Results and Discussions

**FIGURE 2:**
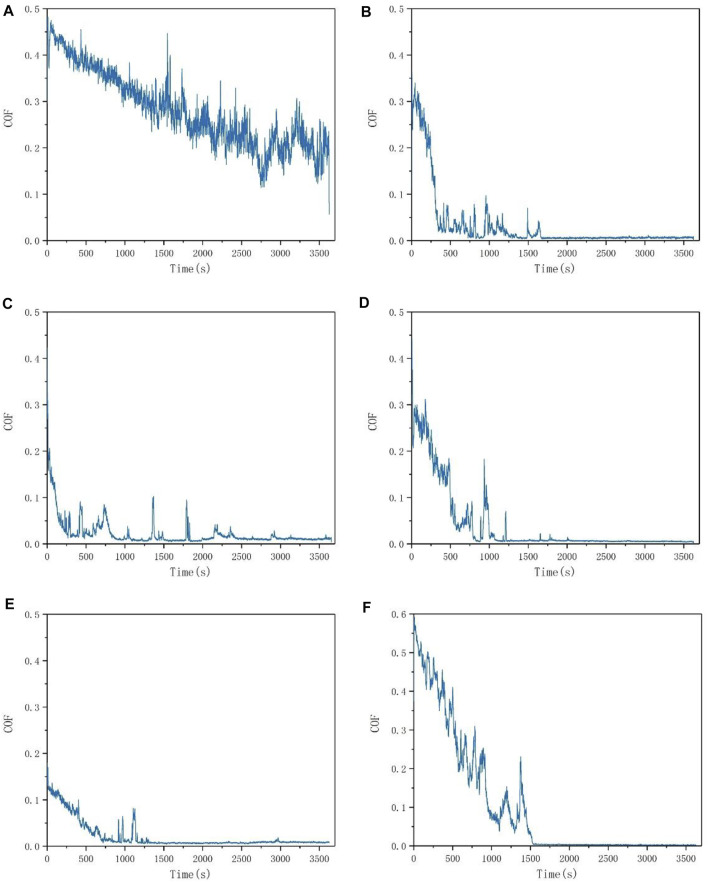
The friction experiment COF curve **(A)** deionized water **(B)** 0.25wt% amino-modified ZIF-8 **(C)** 0.5wt% amino-modified ZIF-8 **(D)** 1wt% amino-modified ZIF-8 **(E)** 2wt% amino-modified ZIF-8 **(F)** 1wt% unmodified ZIF-8.

The friction coefficient curve of deionized water is shown in Figure 2A. The friction coefficient was high during the whole experiment and the running-in was slow with a poor lubrication effect.

When the amino-modified ZIF-8 nanoparticles were added into the deionized water as lubrication additives, the initial friction coefficient visibly decreased. The friction coefficient decreased rapidly in the further phases of the friction process, and the fluctuation of the COF curve was significantly below that of the deionized water. Hence, the friction stability was very good.

Further, aiming to verify the effect of nanoparticle concentration on the solution lubrication property, four amino-modified ZIF-8 nanoparticle solutions with solution concentrations of 0.25wt%, 0.5wt%, 1wt%, and 2wt% were compared. Their lubrication effects were evaluated and the concentration with the optimal lubricating properties was found. The friction coefficient curves are shown in Figure 2B–E. According to our experimental observation, when the concentration exceeded 2wt%, the fluidity of the solution decreased obviously, thus the solution viscosity increased. The friction between friction pairs increased significantly, and the lubrication effect decreased. Therefore, the maximum experimental solution concentration was set to 2wt%.

The initial friction coefficient decreased with the increase of concentration. When the concentration was 2wt%, the initial friction coefficient was approximately 0.15, which might be due to the increase of viscosity. The viscosity increased when the concentration reached a certain value, improving the friction pair lubrication conditions in the initial state, decreasing the initial friction coefficient.

After processing the experiment data and results, outputs are shown in Table 1. The average friction coefficient during the whole process firstly increased, followed by a decrease. The highest value was reached at 1wt%, while the lowest was reached at 2wt%. This phenomenon might be caused by simultaneous increases in viscosity and concentration, which affected the friction coefficient during the running-in period. For this reason, the average friction coefficient was affected during the whole process.

**TABLE 1:** Effects of ZIF-8 nanoparticles on the friction effect for various concentrations.

Concentration	Average COF during the whole process	Running-in time (s)	Smooth friction period COF
0.25wt%	0.0149	794.624	0.0101
0.5wt%	0.0165	824.832	0.0092
1wt%	0.0186	1,070.224	0.0049
2wt%	0.0101	1,142.176	0.0083

With the increase of the concentration, COF in the smooth friction period firstly decreased and then increased. The lowest friction was found for the 1wt% concentration–only 0.0049. This might be due to the gradual increase of the ZIF-8 concentration and the increase of ZIF-8 nanoparticles involved in the friction. Moreover, the amount of ZIF-8 nanoparticles gradually increased from insufficient to excessive. At the same time, the friction surface was no longer unable to form a stable lubricating film. The nanoparticles not only formed a stable lubricating film on the friction surface but also continuously repaired the film during the friction process. Hence, the surface was always in a dynamic stable state.

When the concentration was increased to 2wt%, the friction coefficient suddenly increased in the smooth friction period, which could be caused by the excessive ZIF-8 nanoparticles. The excessive nanoparticles were easy to agglomerate in the solution, increasing the friction resistance and subsequently, the friction coefficient, affecting the lubrication. Moreover, the excessive concentration led to the increase of solution viscosity, also increasing the friction coefficient.

Finally, when the concentration of ZIF-8 nanoparticles was 0.25wt%, the wear of silicon nitride ceramic ball was the highest. In that case, the running-in time was the shortest and the smooth friction period was reached as soon as possible. However, the friction coefficient was high during the whole process, including the smooth friction period. In other words, the lubrication effect was not significant. For comparison, the friction coefficient of 1wt% solution was the lowest during the smooth friction period, and its super lubrication state was better than that of other concentrations. At 2wt%, although the average friction coefficient was the lowest during the whole process, the running-in time was the longest, and the friction coefficient in the smooth friction period was high. It should be noted that the standard for evaluating the optimal concentration is not unique and this standard can be changed according to actual needs. Comprehensively considering average COF during the whole process, running-in time and smooth friction period COF, when the concentration of ZIF-8 nanoparticles with a particle size of 100 nm in an aqueous solution was 1wt%, the lubrication effect was the best. Consequently, the anti-friction and anti-wear effects were the best, making it the optimal concentration.

The unmodified ZIF-8 nanoparticle solution with the concentration of 1wt% was also examined in the tribological experiment to verify the lubrication effect of amino-modified ZIF-8 nanoparticle solution. The COF curve is shown in Figure 2F. It was observed that the friction coefficient of the modified ZIF-8 nanoparticle aqueous solution was lower compared to that of its unmodified counterpart during the running-in period. Its COF was more stable during the whole friction process and the running-in time was shorter. Hence, the friction performance was improved significantly.

The tribological properties of the amino-modified ZIF-8 nanoparticle solutions were compared to those of the deionized water and unmodified solution. The four experimental parameters, wear scar diameter, running-in time, COF in smooth friction period, and the average friction coefficient during the process, were compared. Additionally, their advantages and disadvantages were examined and summarized.

**FIGURE 3:**
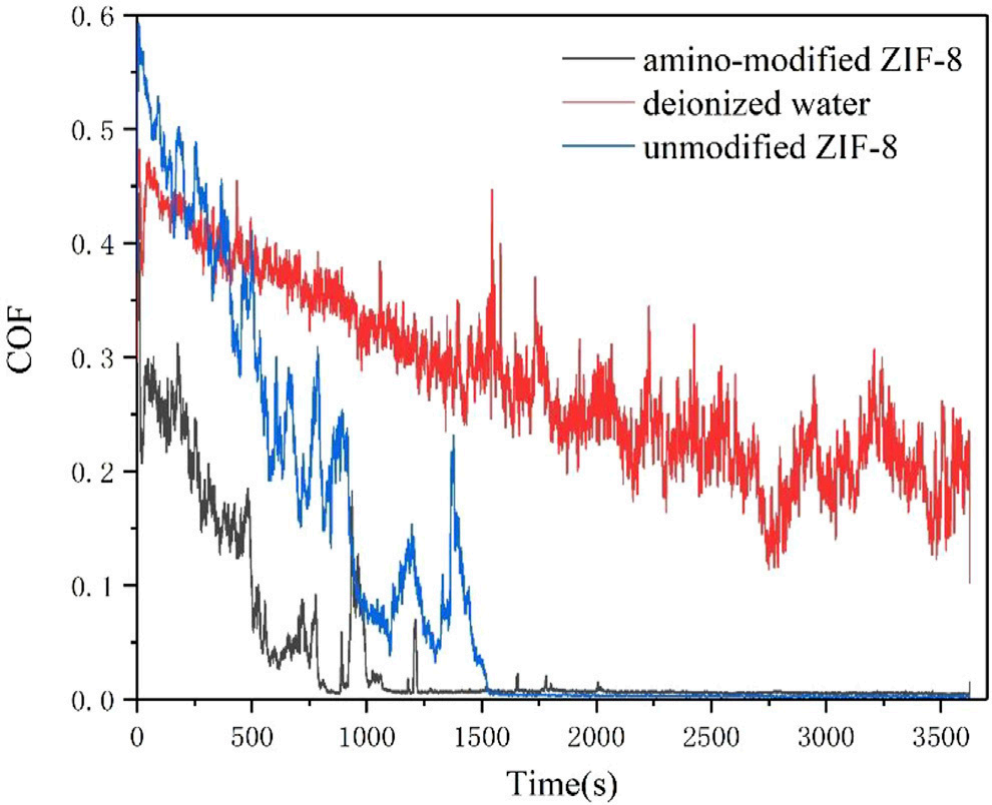
Comparison of COF curves of deionized water, unmodified ZIF-8 nanoparticles and amino-modified ZIF-8 nanoparticles.

According to Figure 3, the running-in time of nanoparticles was shorter than that of deionized water. It could be seen that amino-modified ZIF-8 nanoparticles, as silicon nitride ceramic water lubrication additives, exhibited a good lubrication effect. The running-in time was greatly reduced; therefore, it could reach a stable friction state more quickly. Further, according to the analysis concentrating on COF data, the average friction coefficient of amino-modified ZIF-8 nanoparticles during the stable period was 0.0049, meaning that the stable superlubricity state was reached. In addition, according to the measurement of electron microscope, the wear scar diameter of deionized water, unmodified ZIF-8 nanoparticles and amino-modified ZIF-8 nanoparticles was 1.62, 1.33 and 1.25 mm respectively.

The lubrication effect of deionized water was the worst according to all the criteria-wear scar diameter, average friction coefficient, running in time, and friction coefficient during the smooth friction period. Additionally, the wear of silicon nitride ceramic balls was severe. On the other hand, the modified ZIF-8 nanoparticle solution exhibited the best lubrication properties. Compared to unmodified ZIF-8 nanoparticle solution, its friction period COF was greatly reduced during the running-in time. Although the friction coefficient change was limited during the smooth friction period, the running-in time of amino-modified ZIF-8 nanoparticle solution was shorter than that of unmodified ZIF-8 nanoparticle solution. Therefore, it could be concluded that the lubrication effect of modified ZIF-8 nanoparticle aqueous solution as Si_3_N_4_ ceramic water lubricant was better than that of the unmodified solution.

**FIGURE 4:**
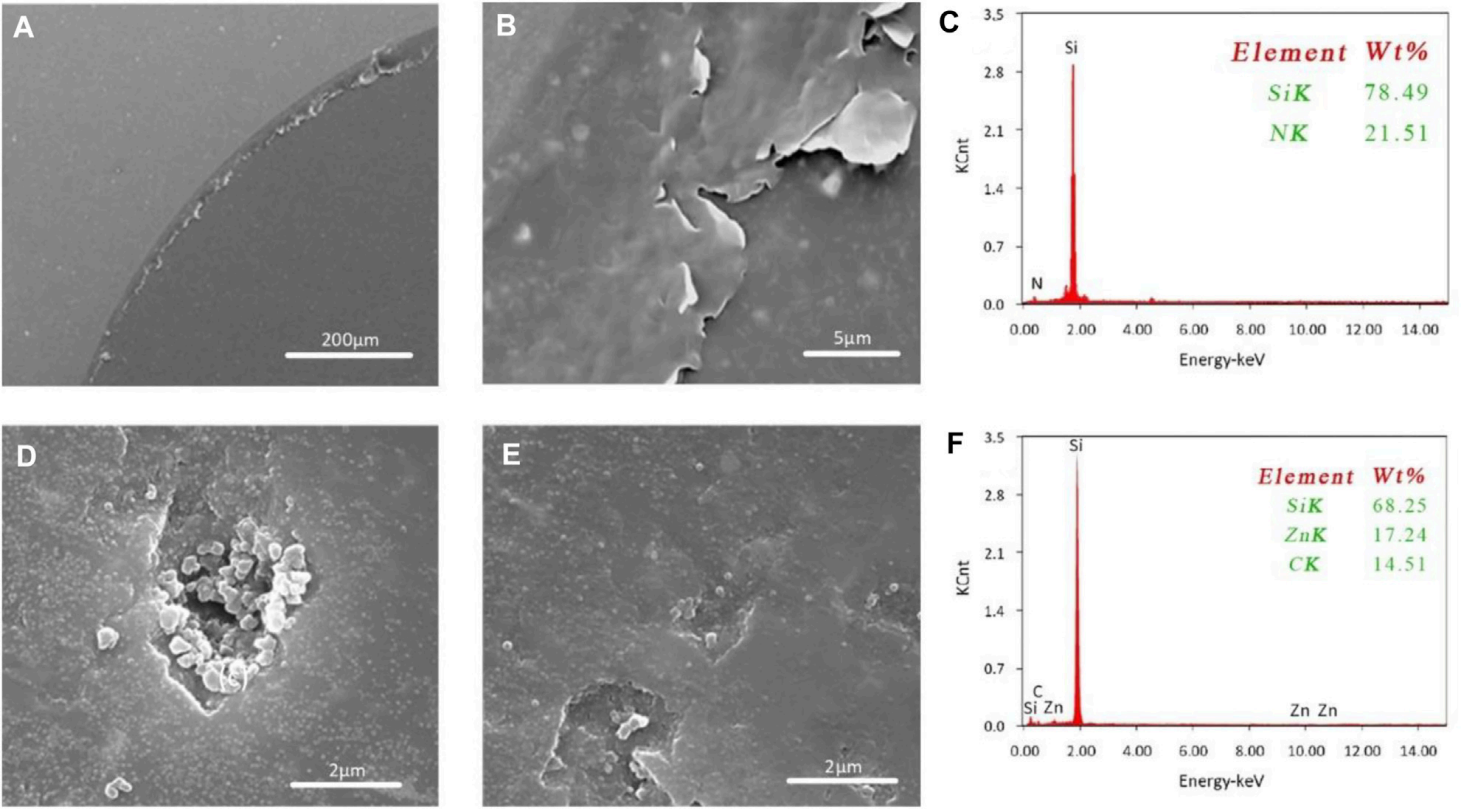
SEM and EDS characterization images of wear spot surface **(A)** water lubrication film forming SEM **(B)** water lubrication film destroying SEM **(C)** EDS before friction **(D)** SEM filling effect **(E)** SEM film-forming effect **(F)** EDS after the friction.

The surface of the silicon nitride ceramic ball wear scar, which was lubricated with amino-modified ZIF-8 nanoparticle solution, was analyzed via SEM and EDS. The results are shown in Figure 4. As shown in Figure 4A, when deionized water was used as a ceramic lubricant, protective film was formed on the friction surface, resulting in a super-lubricant surface (formed due to tribochemical reaction). As shown in Figure 4B, a uniform lubricating film was formed on the friction surface. However, the lubricating film in some areas was peeled off, which may be due to the friction and wear. Due to the existence of the super-lubricant surface and the protective film, deionized water could reach the super-lubricity state when used as a lubricant. Therefore, the film could protect the friction surface and reduce the friction. However, the lubricating film was unstable during the water lubrication, the running-in time was long, and the wear was severe (Xu and Kato, 2020; Li et al., 2021).

Next, the EDS energy spectrum surface analysis was carried out before and after the friction, and the surface elements were analyzed. The results are shown in Figure 4C, F; the highest peak was found for Si, indicating that it represented all the Si elements of silicon nitride ceramic ball. Further, there were several Zn peaks in the film area, along with the C and Si. It showed that the amino-modified ZIF-8 nanoparticles were successfully adsorbed into the wear surface.

Although the silicon nitride ceramic ball surface was macroscopically very smooth after the running-in, through SEM observation it was found that many surface defects remained. These microscopic defects were the main reason preventing the friction coefficient from quickly entering a stable state. However, when the ZIF-8 nanoparticles were used as ceramic water lubricant additives, they would fill the surface defects, further reducing the friction coefficient. Moreover, ZIF-8 nanoparticles located on the friction surface could also cooperate with the silica gel film, improving the surface film stability and the adsorption performance. The combined action of filling and film-forming resulted in a good friction performance, as shown in Figure 4D.

Nanoparticles could improve the friction surface smoothness on the micro-level, improving its friction performance and achieving super-lubricity. Moreover, the filling effect formed a dense protective film on the friction surface, which could both bear the pressure and prevent the surface film from grinding. As shown in Figure 4E, ZIF-8 nanoparticles could be seen below the water lubrication film.

**FIGURE 5:**
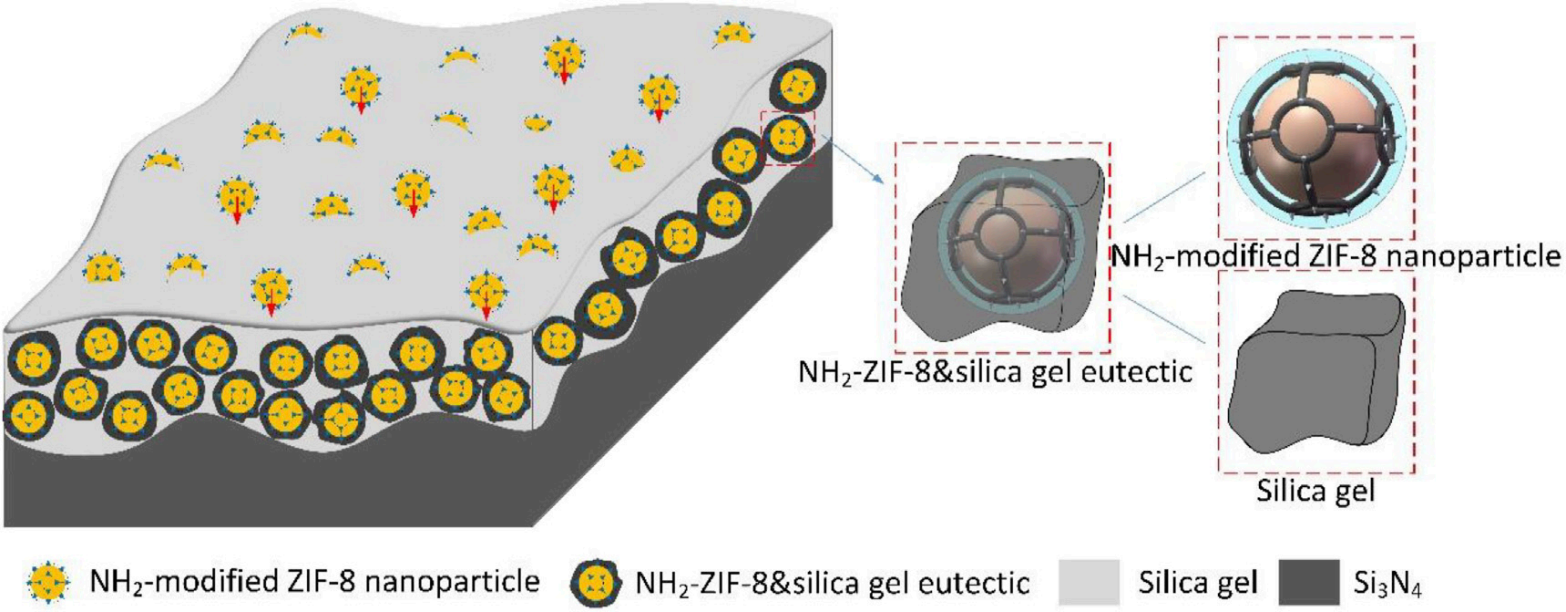
Synergistic film-forming mechanism of ZIF-8 nanoparticles and silica gel.

Based on the previous analysis, the ZIF-8 working mechanism is shown in Figure 5. During the friction process, nanoparticles are buried into the silica gel film produced by tribochemical reaction due to pressure; some nanoparticles are covered on the friction surface. With the formation of water-lubricated silica gel film, the structure of ZIF-8 nanoparticles was fused with the silica gel, forming the NH_2_-ZIF-8&silica gel eutectic. The ZIF-8 supported the surface while and silica gel filled it; therefore, the water lubrication film was strengthened, becoming denser. Moreover, it could be quickly filled when the lubrication film was damaged. Thus, its protective effect was greater than that of water lubrication film. More work and in-depth study should be conducted on this issue to further understand the formation of NH_2_-ZIF-8&silica gel eutectic in future research.

Finally, to conclude, the ZIF-8 nanoparticles could be fused with silica gel to fill the defects on ceramic surfaces. Doing so made the water lubricating film more stable, while also improving the formation of a dense lubricating film on the ceramic surface, which resulted in excellent lubrication performance.

## Conclusion

The influence of ZIF-8 nanoparticles on the water lubrication effect in silicon nitride ceramics was studied in this paper. Based on a series of friction experiments and characterization analysis, the following conclusions were drawn:

(1) When ZIF-8 nanoparticle aqueous solution is used as silicon nitride ceramic water lubricant additive, it exhibits good lubrication performance. Additionally, its lubrication effect is significantly better compared to that of deionized water.
(2) The lubrication performance of amino-modified ZIF-8 nanoparticles solution is better than that of the unmodified solution.
(3) The friction experiments of amino-modified ZIF-8 nanoparticle aqueous solutions with four different concentrations (0.25wt%, 0.5wt%, 1wt%, and 2wt%) were carried out. It was found that the lubrication performance in the smooth friction period was the best (the smooth friction period COF was 0.0049, lowest among all the experimental groups) and the wear scar diameter was the lowest (1.25 mm) when the concentration of amino-modified ZIF-8 nanoparticles was 1wt%. Comprehensively considering running-in time, average COF during the whole process and smooth friction period COF, when the concentration of ZIF-8 nanoparticles was 1wt%, the lubrication effect was the best.
(4) The ZIF-8 nanoparticles can improve the lubrication property of silicon nitride ceramics mainly through the following two mechanisms - filling and film-forming. The nanoparticles can be fused with silica gel to improve the water lubrication film stability and lubrication performance.

## Data Availability

The original contributions presented in the study are included in the article/supplementary material, further inquiries can be directed to the corresponding author.
